# Evaluation of the Response of HOS and Saos-2 Osteosarcoma Cell Lines When Exposed to Different Sizes and Concentrations of Silver Nanoparticles

**DOI:** 10.1155/2021/5013065

**Published:** 2021-12-13

**Authors:** Konstantinos Michalakis, Athina Bakopoulou, Eleni Papachristou, Dimitra Vasilaki, Alexandros Tsouknidas, Nikolaos Michailidis, Elaine Johnstone

**Affiliations:** ^1^School of Health Sciences, Aristotle University of Thessaloniki, Thessaloniki, Greece; ^2^Tufts University, Boston, MA, USA; ^3^University of Oxford, Oxford, UK; ^4^Laboratory for Biomaterials and Computational Mechanics, Department of Mechanical Engineering, University of Western Macedonia, Kozani, Greece; ^5^Department of Mechanical Engineering, School of Engineering, Aristotle University of Thessaloniki, Thessaloniki, Greece; ^6^Department of Oncology, University of Oxford, Oxford, UK

## Abstract

Osteosarcoma is considered to be a highly malignant tumor affecting primarily long bones. It metastasizes widely, primarily to the lungs, resulting in poor survival rates of between 19 and 30%. Standard treatment consists of surgical removal of the affected site, with neoadjuvant and adjuvant chemotherapy commonly used, with the usual side effects and complications. There is a need for new treatments in this area, and silver nanoparticles (AgNPs) are one potential avenue for exploration. AgNPs have been found to possess antitumor and cytotoxic activity in vitro, by demonstrating decreased viability of cancer cells through cell cycle arrest and subsequent apoptosis. Integral to these pathways is tumor protein p53, a tumor suppressor which plays a critical role in maintaining genome stability by regulating cell division, after DNA damage. The purpose of this study was to determine if p53 mediates any difference in the response of the osteosarcoma cells in vitro when different sizes and concentrations of AgNPs are administered. Two cell lines were studied: p53-expressing HOS cells and p53-deficient Saos-2 cells. The results of this study suggest that the presence of protein p53 significantly affects the efficacy of AgNPs on osteosarcoma cells.

## 1. Introduction

**Figure 1:**
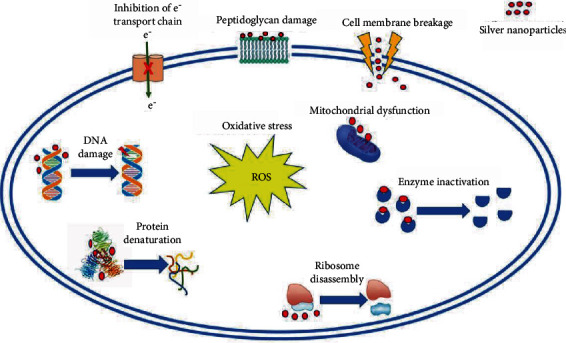
Mechanisms of action of AgNPs on cells.

**Figure 2:**
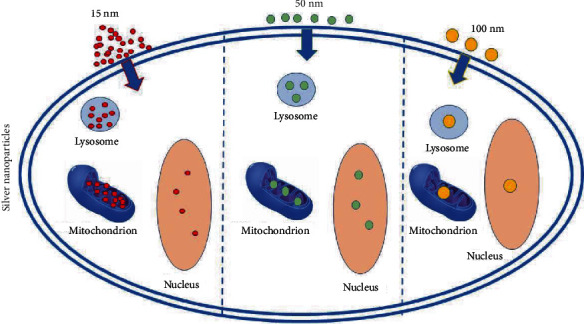
Efficacy of AgNPs according to their size.

Osteosarcoma is considered a relatively uncommon malignant disease. Nevertheless, it is the most common cancer arising from bone [1]. It usually affects adolescents and young adults. In recent years, much advancement has been made in treating osteosarcoma, which combines surgery, chemotherapy, and sometimes radiotherapy. Currently, the 5-year survival rate for patients diagnosed with osteosarcoma is 60-70% [2–5]. The chemotherapy agents employed include cisplatin, doxorubicin, ifosfamide, and methotrexate. Other cytotoxic agents such as etoposide and different combinations have also been suggested in the literature [6]. Nevertheless, the use of these drugs has several side effects and complications including neutropenia, mouth ulcers, fatigue, severe diarrhea, nausea, and vomiting. The side effects can be very serious and commonly require hospitalization. Cardiomyopathies and irreversible lung fibrosis have also been described, illustrating that severe side effects present a major drawback for the use of chemotherapeutic agents [7]. This along with therapeutic limitations, due to the systemic cytotoxic effects, has motivated scientists to start exploring different directions in an attempt to find innovative therapies for several types of cancer, including osteosarcoma [8–15]. Some novel therapeutic agents have been tested for that purpose, including tumor microenvironment inhibitors, which target signal-transduction pathways and immunomodulatory agents. Methods for overcoming resistance mechanisms as well as new delivery mechanisms have also been tested [16]. One of these avenues of interest is silver nanoparticles. Although the exact action by which AgNPs act on cells is not fully understood, it is speculated that a Trojan horse mechanism is involved [17]. Upon entering the cell, the AgNPs release silver ions in the cytoplasm which then induce the formation of ROS, thus causing an imbalance of the cell's redox homeostasis [18, 19]. It is not known yet whether the observed oxidative damage is due to the action of AgNPs per se, accumulation of silver ions in the cytoplasm, or a combination of both [20, 21] (Figure 1). A recent in vitro study testing the antibacterial effect of AgNPs with different sizes has shown that smallest-sized AgNPs are more efficacious on two different types of Gram-negative bacteria [22]. According to Gliga et al., smaller AgNPs are more active due to the increased Ag ion release from the increased total surface area [23] (Figure 2).

Tumor protein p53, whose gene TP53 is located on the short arm of chromosome 17, plays a critical role in regulating cell division, after DNA damage occurs. It is crucial in determining if the DNA damage can be repaired or if the cell will undergo apoptosis [24, 25]. When DNA damage in the form of a double-strand break occurs, there is recruitment of ATM serine protein kinases and/or ATR kinases, which are then activated. These kinases phosphorylate p53, leading the protein to evade degradation by ubiquitin. As a result, the levels of p53 increase markedly; the protein is stabilized and activates transcription of p21(Cip1/Waf1) [26]. The latter acts by binding and inhibiting the activity of several complexes, including cyclin E-CDK2, cyclin E-CDK1, and cyclin E-CDK4/6, and prevents cell cycle progression at phase G_1_ [27, 28]. This arrest gives time to the cell to repair the damage of the DNA. Furthermore, p53 is responsible for the production of DNA repair enzymes and proapoptotic proteins [29].

In this way, p53 acts as a tumor suppressor, and its inactivation seems to play a key role in the development of human cancer. For the pivotal role in maintaining genome integrity, p53 has been named “guardian of the genome” [30]. If DNA is damaged and p53 is present and functional, the cell cycle arrests in phase G_1_. On the contrary, in the absence of functional p53, cells continue to grow and divide. The p53 protein is unique in the sense that it exists in very small quantities in normal cells, due to its instability and rapid degradation. Mouse models have shown that the absence of p53 is associated with the development of several types of tumors [31]. Furthermore, p53 is mutated in more than half of all human cancers, and in more than 80% of tumors, there is a p53 signaling pathway disruption of some kind [32–34].

Several human osteosarcoma cell lines have been isolated so far, including the HOS, U-2OS, MG-63, G-292, and Saos-2. An analysis of these cell lines with p53 genomic probes has revealed some key differences. p53 was found to be present in G-292, MG-63, HOS, and U-2OS cell lines, with a rearrangement in the first intron of the gene described in G-292 and MG-63. A point mutation within the p53 coding sequence has been described in HOS cells which results in overproduction of mutant p53 [35, 36].

There has been speculation that the AgNP-induced mechanism of cytotoxicity may be affected by the presence of functional p53 [37], although the evidence is rather limited. Therefore, the possible differences in the effect that AgNPs have on the viability of different human osteosarcoma cell lines, in which p53 is expressed or not, should be further investigated. The aim of this study was to determine if there are any differences in the response of two cell lines: p53-expressing HOS cells and p53-deficient Saos-2 cells, after different sizes and concentrations of AgNPs are administered. The null hypotheses were the following:

- The size of AgNPs would not affect the response of p53-expressing HOS cells and p53-deficient Saos-2 osteosarcoma cell lines
- The AgNP content of the colloid would not affect the response of p53-expressing HOS cells and p53-deficient Saos-2 osteosarcoma cell lines
- The presence or absence of p53 would not affect the response of osteosarcoma cells to AgNP treatment

## 2. Methods and Materials

### 2.1. Osteosarcoma Cells

**Figure 3:**
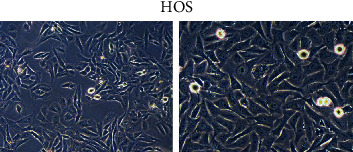
HOS (p53-expressing) osteosarcoma cells used for the purposes of this study.

**Figure 4:**
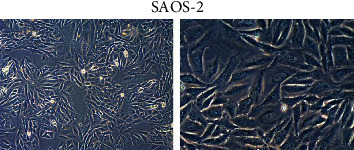
Saos-2 (p53-deficient) osteosarcoma cells used for the purposes of this study.

The HOS (p53-expressing) (Figure 3) and Saos-2 (p53-deficient) (Figure 4) cells needed for this research project were obtained from the American Type Culture Collection (ATCC No. HTB 85).

### 2.2. Silver Nanoparticle Preparation

**Figure 5:**
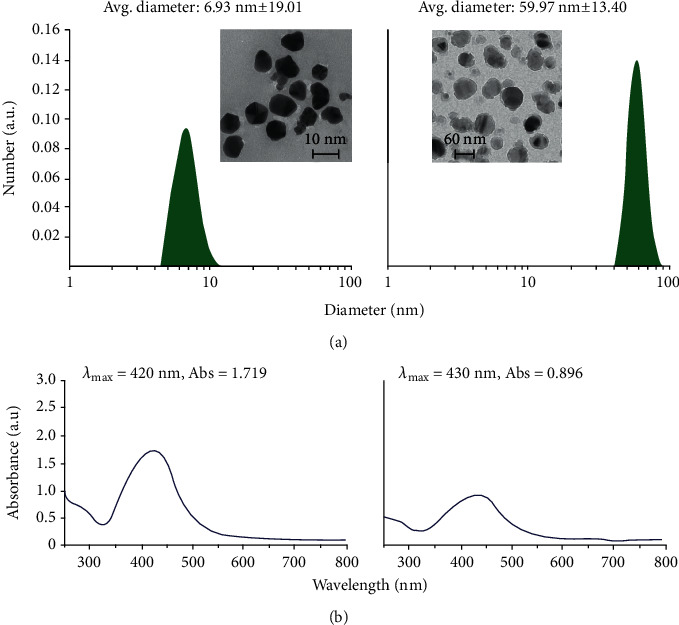
Characteristics of the colloidal silver suspensions, namely, (a) size distribution and indicative TEM image (for the 7 nm colloid) and (b) UV-Vis spectra.

Two commercially available colloidal suspensions (PLiN Nanotechnology) with monodispersed populations of spherical AgNPs, i.e., 7 nm and 60 nm in size, respectively, were synthesized as summarized below. Silver nitrate (99.9% AgNO_3_, Mr = 169.873 g/mol) was used as a silver precursor (Duchefa Biochemie) for the reduction into AgNPs, with components conventionally found in literature, while a protein with a molecular mass of 20-25 kg/mol (Sigma-Aldrich) was employed as the stabilizer. AgNPs were produced via liquid chemistry, by adding the reduction agent to the preheated aqueous solution of the silver nitrate, stirred along with the stabilizer, to ensure complete dissolution. The characteristics of the AgNPs are presented in Table 1 and Figure 5.

p53-expressing HOS and p53-deficient Saos-2 osteosarcoma cells were treated with colloid silver (PLiN Nanotechnology, Thessaloniki, Greece) of 7 nm and 60 nm positively charged AgNPs, as determined by dynamic light scattering. In addition to control (c), which contained no AgNPs, six different concentrations (c1-c6) were tested for three time periods, i.e., 24, 48, and 72 hours. These concentrations were as follows: c1 = 10 ppm, c2 = 5 ppm, c3 = 2.5 ppm, c4 = 1.25 ppm, c5 = 0.625 ppm, and c6 = 0.3125 ppm [37].

### 2.3. HOS and Saos-2 Cell Culture

HOS and Saos-2 cells were expanded in cell culture media (CCM) in 75 cm^2^ flasks. Cell cultures were maintained in an incubator at 37°C, in 5% CO_2_ and 95% humidity until reaching 80-90% confluency. Cell harvesting from the flask surface was performed using 0.25% Trypsin/1 mM EDTA solution (Invitrogen). For cell counting and determination of cell density and percentage of dead cells before each experimental assay, an improved Neubauer hemocytometer (Laboroptik, Lancing, UK) and Trypan blue exclusion tests were used [38].

### 2.4. Evaluation of Cell Viability with the MTT Assay

The viability of HOS and Saos-2 cells was investigated by the MTT (3-(4,5-dimethylthiazol-2-yl)-2,5-diphenyltetrazolium bromide) assay. Cells were cultured in direct contact with the specimens in 96-well plates (10^4^ cells/well) for 24, 48, and 72 h, at 37°C and 5% CO_2_. After these three time points, MTT (5 mg/ml in CCM) was added to each well containing the specimens, and the plates were incubated for 4 h at 37°C and 5% CO_2_. During this period, the NAD(P)H-dependent cellular oxidoreductase enzymes of mitochondria reduce the tetrazolium dye MTT to its insoluble formazan, which has a purple color. After this period, the medium containing the MTT solution was discarded and 500 *μ*l of DMSO (dimethyl sulfoxide) was added to each well and incubated for 1 h at 37°C to dissolve the insoluble purple formazan product into a colored solution. Then, the optical density (OD) was measured against blank (DMSO), at a wavelength of 545 nm and a reference filter of 630 nm by a microplate reader (Epoch, Biotek, Biotek Instruments, Inc., Vermont, USA). The experiments were repeated three times, with 6-8 replicates for each repetition. All results were expressed as an average percentage of the control value [38].

### 2.5. Evaluation of Cell Proliferation with the BrdU Assay

The proliferation rates of HOS and Saos-2 cells seeded of each group were investigated by the BrdU (5-bromo-2′-deoxyuridine) assay (Sigma-Aldrich, Roche Diagnostics, Manheim, Germany).

Cells were cultured in 96-well plates (10^4^ cells/well) for 24, 48, and 72 h, at 37°C and 5% CO_2_, as described earlier. Afterwards, BrdU was added at a concentration of 10 *μΜ*, and the plates were incubated for 6 h at 37°C and 5% CO_2_. Then, treated cells were fixed with FixDenat® solution (at 15-25°C, for 30 min), according to the manufacturer's recommendations, and exposed to a peroxidase-conjugated BrdU antibody (anti-BrdU-POD) at a concentration of 10 *μΜ* for 90 min. Afterwards, 200 *μ*l of 3-3′-5-5′-tetra-methyl-benzidine substrate (TMB) was added to each well. The blue color peroxidase-substrate reaction ended after 5 min, by an H_2_SO_4_ solution (stop solution, 50 *μ*l/well). The incorporated BrdU were quantified by measuring the OD in a microplate reader (Epoch, Biotek, Biotek Instruments, Inc., Vermont, U.S.A.), at a wavelength of 450 nm and a reference filter of 690 nm. Cell-free and BrdU-free wells served as internal controls for this assay. The resulting OD values of those wells were used as blank (negative control) and background control (positive control), respectively. The experiments were repeated three times, with 6-8 replicates for each repetition. All results were expressed as an average percentage of the control value [38].

**Figure 6:**
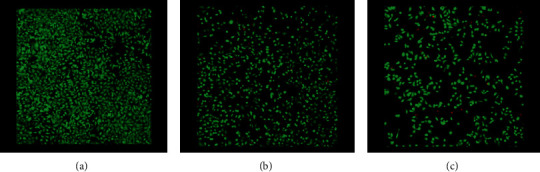
Live/dead double staining detecting viable and dead HOS cells when exposed to 2.5 ppm concentration AgNPs, after 48 hours: (a) control, (b) 7 nm, and (c) 60 nm (magnification ×100).

**Figure 7:**
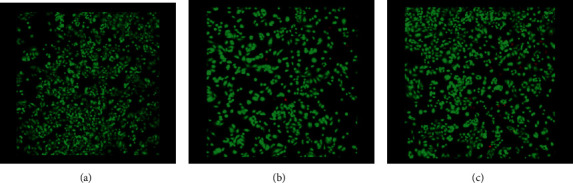
Live/dead double staining detecting viable and dead Saos-2 cells when exposed to 2.5 ppm concentration AgNPs, after (a) control, (b) 7 nm, and (c) 60 nm (magnification ×100).

Live/dead double staining was utilized to detect viable and dead Saos-2 and HOS cells when exposed to AgNPs. Calcein-AM, which is a highly lipophilic and cell membrane-permeable dye, and the nuclei-staining dye Propidium Iodine, which cannot pass through a viable cell membrane, were utilized for that purpose. A 490 nm light was used for simultaneous monitoring of viable and dead cells with a single-excitation fluorescence microscope (Figures 6 and 7).

### 2.6. Statistical Analysis

For statistical analysis, Prism 6 (GraphPad, CA, U.S.A.) software was utilized. A two-way Analysis of Variance (ANOVA) was performed for the viability assays, while for follow-up comparisons between groups and time points, Tukey's post hoc test was employed. Normal distribution was confirmed by Kolmogorov-Smirnov normality tests. The level of statistical significance was set to 0.05 (*α* = 0.05).

## 3. Results

### 3.1. Evaluation of Cell Viability by the MTT Assay

HOS (p53-expressing) and Saos-2 (p53-deficient) osteosarcoma cell viability was assessed by the MTT assay, for two different AgNP sizes (7 and 60 nm) and six different concentrations (c1 = 10 ppm, c2 = 5 ppm, c3 = 2.5 ppm, c4 = 1.25 ppm, c5 = 0.625 ppm, and c6 = 0.3125 ppm), at three time points (24 hours, 48 hours, and 72 hours).

### 3.2. Evaluation of HOS Cell Viability for 7 nm and 60 nm AgNPs

#### 3.2.1. 7 nm

The 10 ppm and the 5 ppm concentrations of the 7 nm AgNPs demonstrated a remarkably decreased metabolic activity for all three time points which was statistically significant (*P* < 0.0001).

**Figure 8:**
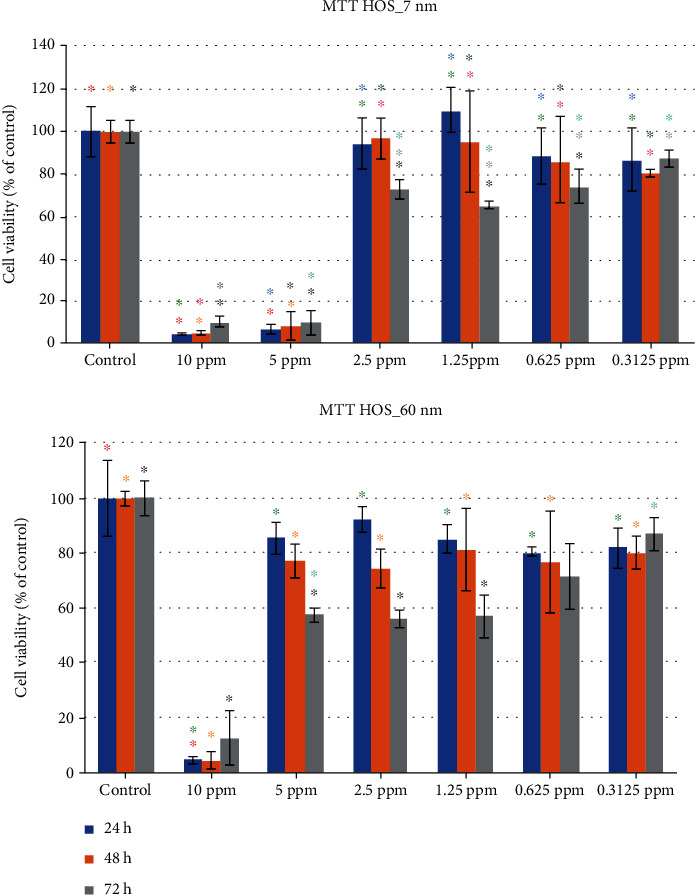
HOS cell viability (percentage values), when subjected to exposure of different concentrations of 7 nm and 60 nm AgNPs (same color asterisks indicate statistically significant differences between the control and suspensions of different AgNP concentrations. Absence of asterisks or absence of same color asterisks indicates no statistically significant differences, according to Tukey's HSD test for *α* = 0.05).

At lower concentrations, a small increase in viability was observed. The biggest increase in cell viability (110% (±10.29%)) was noticed in 24 hours at the 1.25 ppm concentration. The smallest increase (65.58% (±1.86%)) was also noticed at the 1.25 ppm concentration, in 72 hours (Table 2 and Figure 8).

#### 3.2.2. 60 nm

Unlike the 7 nm AgNPs, in the 60 nm AgNPs, only the 10 ppm concentration demonstrated a remarkably decreased cell viability, for all three examined time periods. Specifically, these values were 5% (±1.20%), 4.84% (±3.05%), and 12.88% (±9.85%) at the 24-hour, 48-hour, and 72-hour time periods, respectively. A big increase in cell viability was noticed for all other concentrations, ranging between 56.13% (±3.23%) and 92.33% (±4.65%). The smallest value was noticed at 72 hours, while the biggest one at 24 hours, both in the 2.5 ppm concentration (Table 2 and Figure 8).

The 2-way ANOVA (*α* = 0.05) for the MTT assay of HOS cells subjected to 7 nm AgNPs revealed a statistically significant effect of the concentration factor (*F* = 5.618, *P* = 0.0069), the time factor (*F* = 144.3, *P* < 0.0001), and their interaction (*F* = 2.869, *P* = 0.0057), while for the 60 nm AgNPs, the ANOVA revealed a statistically significant effect of the concentration factor (*F* = 12.30, *P* < 0.0001), the time factor (*F* = 112.5, *P* < 0.0001), and their interaction (*F* = 3.976, *P* = 0.0004).

### 3.3. Evaluation of Saos-2 Cell Viability for 7 nm and 60 nm AgNPs

An entirely different behavior of the Saos-2 osteosarcoma cells is observed when compared to the HOS cells.

#### 3.3.1. **7** nm

**Figure 9:**
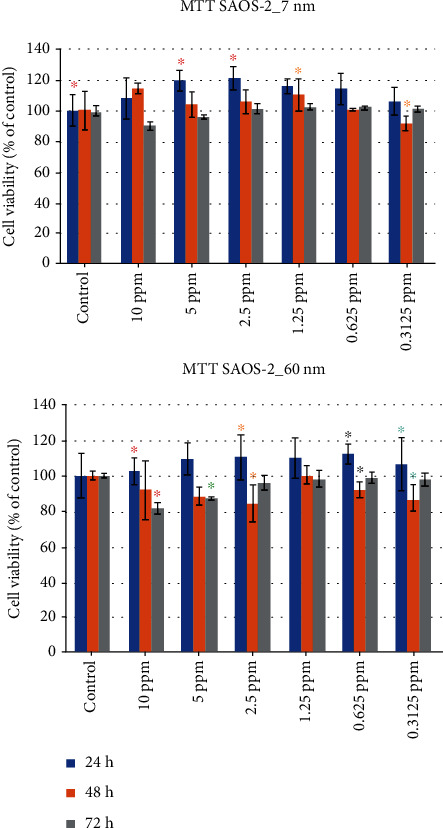
Saos-2 cell viability (percentage values), when subjected to exposure of different concentrations of 7 nm and 60 nm AgNPs (same color asterisks indicate statistically significant differences between the control and suspensions of different AgNP concentrations. Absence of asterisks or absence of same color asterisks indicates no statistically significant differences, according to Tukey's HSD test for *α* = 0.05).

The smallest percentage in cell viability was 90.03% (±2.50%), noticed at 72 hours in the 10 ppm concentration, while the biggest one was 121.35% (±7.42%). The latter was observed at 24 hours in the 2.5 ppm concentration. In general, very little cytotoxicity was observed at all concentrations and time points (Table 3 and Figure 9).

#### 3.3.2. 60 nm

Similarly, with the 7 nm AgNPs, the 60 nm AgNPs at 10 ppm did not produce a decreased Saos-2 cell viability for any concentration, when compared to the control. In general, it can be observed that in the 24-hour period, all concentrations did not have a negative effect on Saos-2 cells. On the opposite, it seems that it promoted cell viability, as it reached 112.28% (±5.57%), for the 0.625 ppm concentration (Table 3 and Figure 9).

The 2-way ANOVA (*α* = 0.05) for the MTT assay of Saos-2 cells subjected to 7 nm AgNPs revealed a statistically significant effect of the concentration factor (*F* = 18.10, *P* < 0.0001), the time factor (*F* = 3.005, *P* = 0.0156), and their interaction (*F* = 2.690, *P* = 0.0088), while for the Saos-2 cells subjected to 60 nm AgNPs, the ANOVA revealed a statistically significant effect of the concentration factor (*F* = 21.17, *P* < 0.0001). However, a statistically significant effect was not demonstrated for the time factor (*F* = 1.899, *P* = 0.1036) and the interaction of concentration and time (*F* = 1.476, *P* = 0.1720).

### 3.4. Evaluation of Cell Proliferation by the BrdU Assay

HOS (p53-expressing) and Saos-2 (p53-deficient) osteosarcoma cell proliferation was assessed by the BrdU test, for the two different AgNP sizes (7 and 60 nm), six different concentrations (c1 = 10 ppm, c2 = 5 ppm, c3 = 2.5 ppm, c4 = 1.25 ppm, c5 = 0.625 ppm, and c6 = 0.3125 ppm), and three time periods (24 h, 48 h, and 72 h).

### 3.5. Evaluation of HOS Cell Proliferation for 7 nm and 60 nm AgNPs

#### 3.5.1. 7 nm

**Figure 10:**
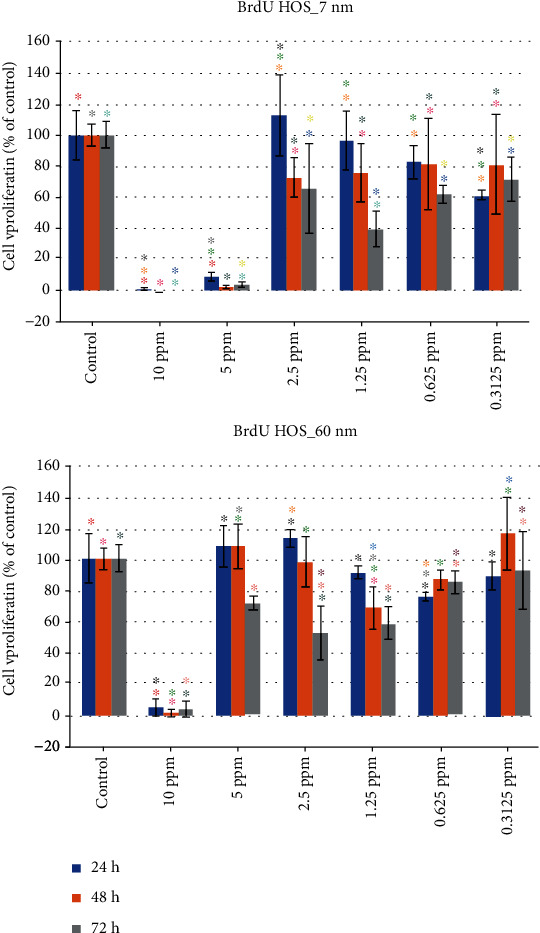
HOS cell proliferation (percentage values), when subjected to exposure of different concentrations of 7 nm and 60 nm AgNPs (same color asterisks indicate statistically significant differences between the control and suspensions of different AgNP concentrations. Absence of asterisks or absence of same color asterisks indicates no statistically significant differences, according to Tukey's HSD test for *α* = 0.05).

Like in the MTT cell viability assay, the 10 ppm and the 5 ppm concentrations of the 7 nm AgNPs demonstrated a remarkably decreased cell proliferation activity for all three time periods, when the BrdU assay was performed (Table 4 and Figure 10).

#### 3.5.2. 60 nm

Unlike the 7 nm AgNPs, in the 60 nm AgNPs, only the 10 ppm concentration demonstrated a remarkably decreased cell proliferation, for all three examined time periods. Specifically, these values were 5.04% (±5.51%), 1.65% (±2.45%), and 4.29% (±4.81%) at the 24-hour, 48-hour, and 72-hour time periods, respectively. A big increase in cell viability was noticed for all other concentrations (Table 4 and Figure 10).

The 2-way ANOVA (*α* = 0.05) for the BrdU assay of HOS cells subjected to 7 nm AgNPs revealed a statistically significant effect of the concentration factor (*F* = 6.534, *P* = 0.0034), the time factor (*F* = 56.95, *P* < 0.0001), and their interaction (*F* = 2.428, *P* = 0.0169), while for the BrdU assay of HOS cells subjected to 60 nm AgNPs, the ANOVA revealed a statistically significant effect of the concentration factor (*F* = 13.14, *P* < 0.0001), the time factor (*F* = 72.19, *P* < 0.0001), and their interaction (*F* = 4.573, *P* = 0.0001).

### 3.6. Evaluation of Saos-2 Cell Proliferation for 7 nm AgNPs

**Figure 11:**
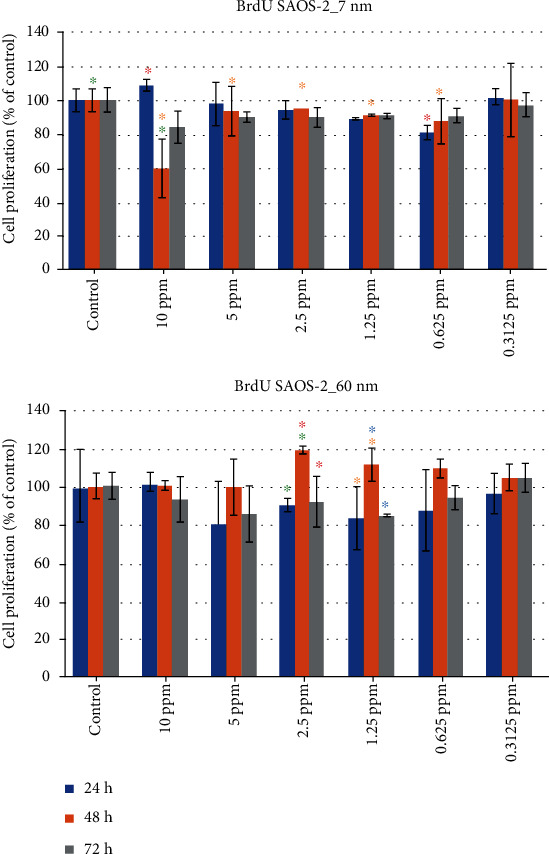
Saos-2 cell proliferation (percentage values), when subjected to exposure of different concentrations of 7 nm and 60 nm AgNPs (same color asterisks indicate statistically significant differences between the control and suspensions of different AgNP concentrations. Absence of asterisks or absence of same color asterisks indicates no statistically significant differences, according to Tukey's HSD test for *α* = 0.05).

An entirely different behavior of the Saos-2 osteosarcoma cells is observed when compared to the HOS cells. While cell proliferation values for the HOS cell ranged between -0.69% and 9.20%, for the examined time periods of the 10 ppm and 5 ppm concentrations, the corresponding values for the Saos-2 cells were 83.93% (±9.43%) and 97.73% (±12.41%) (Table 5 and Figure 11).

The 2-way ANOVA (*α* = 0.05) for the BrdU assay of Saos-2 cells subjected to 7 nm AgNPs did not reveal a statistically significant effect of the concentration factor (*F* = 2.762, *P* = 0.0746). However, a statistically significant effect was demonstrated for the time factor (*F* = 3.936, *P* = 0.0033) and the interaction of concentration and time (*F* = 3.453, *P* = 0.0014). For the BrdU assay of Saos-2 cells subjected to 60 nm AgNPs, the 2-way ANOVA (*α* = 0.05) revealed a statistically significant effect of the concentration factor (*F* = 10.23, *P* = 0.0002). However, a statistically significant effect was not demonstrated neither for the time factor (*F* = 1.481, *P* = 0.2081) nor for the interaction of concentration and time (*F* = 1.219, *P* = 0.3025).

## 4. Discussion

The objective of this study was to investigate if AgNPs of different sizes and concentrations could have a potential application in osteosarcoma treatment and if cytotoxic efficacy was affected by the presence or absence of p53. Although there is published evidence that AgNPs can be used effectively against certain types of osteosarcoma cells [37], there is no study to the authors' knowledge, determining the efficacy of AgNPs against HOS osteosarcoma cells, which express p53 protein.

Two methods, targeting different biological endpoints, were selected to evaluate the impact of AgNPs on the p53-expressing HOS and p53-deficient Saos cell lines. The MTT assay is a typical method to assess cell viability through the evaluation of the active metabolic activity of living cells, whereas the BrdU assay is used for evaluating cell proliferation through DNA intercalation. The combination of the two methods can answer the question of whether a reduction in the metabolic activity observed through the MTT assay is primarily caused by cell death or by cell cycle delays leading to reduced cell proliferation. The latter is a common mechanism of action of several antineoplastic drugs that primarily act by causing cell cycle arrest in different phases of the cell cycle (G1, S, or G2). Based on the above, our main goal was to evaluate such a potential mechanism and balance between cell death and cell cycle delays—which shows an effort of the cell to repair the damage—while morphological observations were performed through phase-contrast microscopy, showing a typical rounding and detachment of the cells at the higher NP concentrations. A range of concentrations between 0.3125 ppm and 10 ppm of both 7 nm and 60 nm AgNPs were tested for effects on cell viability and proliferation in p53-expressing HOS and p53-deficient Saos-2 osteosarcoma cell lines. The concentrations used in this study were selected to align with those used in the main comparatory study [32]. The sizes of AgNPs used in the present study, 7 nm and 60 nm, were selected to be in accordance with other studies reporting on that subject [37, 39, 40].

The results of the present study indicate that all three null hypotheses have to be rejected, as it was demonstrated that the size of AgNPs affected the response of the tested human osteosarcoma cell lines, the concentration of AgNPs affected the response of the tested human osteosarcoma cells, and finally the presence of protein p53 affected the response of osteosarcoma cells to AgNP treatment. However, the results indicate that the size of the AgNPs and the presence of p53 seem to have a stronger impact on the fate of osteosarcoma cells, as a dose response with the concentrations used in this study was not defined.

### 4.1. Effects of Size and Concentration on AgNPs on Cell Viability

The first two hypotheses of this study examined the effect of size and concentration of AgNPs on osteosarcoma cells.

The findings of this study, regarding cell viability, are in partial agreement with those of Kovacs et al. [37]. Our results have clearly demonstrated that the 7 nm AgNPs are very effective in significantly lowering the cell viability of the p53-expressing HOS osteosarcoma cells, at both the 10 ppm and 5 ppm concentrations, at all time points. The same efficacy was not observed at lower concentrations. Kovacs and coworkers found that smaller AgNPs (5 nm) had a stronger cytotoxic effect on wild-type p53-containing U2Os and p53-deficient Saos-2 osteosarcoma cells than larger AgNPs (35 nm) [37]. According to additional published data, there is a faster cellular uptake of smaller rather than larger nanoparticles [41]. Moreover, another study has shown that small AgNPs present a large total surface area and demonstrate greater cytotoxicity, due to the increased release of silver ions [23]. According to that study, the higher silver release is associated with higher cytotoxicity in eukaryotic cells, a finding which was verified by the results of the present study, as well.

In the present study, the 60 nm AgNPs were effective at the 10 ppm concentration, at all time points, while these AgNPs at lower concentrations did not display marked cytotoxicity against the HOS osteosarcoma cells. Unlike the p53-expressing HOS cells, the cell viability of the p53-deficient Saos-2 cells was not markedly affected by the AgNPs used in this study, at any time point, a finding which is not in agreement with the results of Kovacs and coworkers [37]. The results of the present study indicate that only the 7 nm AgNPs at 10 ppm and 5 ppm and the 60 nm AgNPs at 10 ppm reach the threshold toxicity, which significantly lowers the metabolic activity of the HOS osteosarcoma cells [23, 42]. This effect of AgNPs may be explained by differential cellular uptake. It has been previously documented that AgNPs can be incorporated into eukaryotic cells via endocytosis mediated by caveolae and clathrin [43–45]. Furthermore, scanning electron microscopy has verified the presence of AgNPs on cell membranes [46]. However, AgNPs have not been detected either in the nucleus or in the mitochondria [37]. Nevertheless, the endocytosed AgNPs, according to many authors, act as “Trojan horses” carrying and delivering silver ions into the cells [17, 41, 47]. It has been hypothesised that these ions are responsible for all the biological phenomena observed.

All p53-expressing HOS cell groups treated with 7 nm AgNPs at concentrations ranging between 2.5 ppm and 0.3125 ppm or 60 nm AgNPs at concentrations ranging between 5 ppm and 0.3125 ppm revealed an enhanced metabolic activity. The same finding was observed for p53-deficient Saos-2 cell groups treated with 7 nm and 60 nm AgNPs at all tested concentrations. This could be explained by elevated mitochondrial biogenesis, perhaps induced by the oxidative stress that the endocytosed Ag ions caused [48]. Cells suffer from oxidative stress when the cell cannot detoxify and inactivate the reactive oxygen species (ROS) that are produced [49]. It has been well documented in the past that the production of ROS in the mitochondria is a physiological process with ROS being a natural byproduct of oxidative phosphorylation. The electron transport chain on the inner mitochondrial membrane involves ATP synthase and complexes I-IV. Eighty percent of the superoxide, which is produced by complexes I and III, is released into the intermembranous space, while the remaining 20% is released at the mitochondrial matrix [50]. From the interior of the mitochondria, superoxide leaks to the cytoplasm due to the mitochondrial permeability transition pore (mPTP), which is a protein existing in the mitochondrial outer membrane [51, 52]. Subsequently, superoxide dismutase (SOD) catalyzes the partitioning of superoxide to O_2_ and H_2_O_2_ (hydrogen peroxide). This process can take place either in the mitochondrial matrix, where it is catalyzed by MnSOD, or in the cytosol, where it is catalyzed by Cu/ZnSOD. Hydrogen peroxide is considered a highly diffusible second messenger. Crucially, the behavior of tumor cells is influenced by the signaling events which are related to oxidation stress [53–55]. Several events in cancer cell biology are associated with ROS, including adhesion, angiogenesis, survival and apoptosis, metabolism, progression, proliferation, motility, and tumor stemness [56].

### 4.2. Presence of Protein p53 and Cell Viability

The third hypothesis of this study was to determine if the presence of protein p53 affects the response of osteosarcoma cells to treatment with AgNPs.

Many published papers have demonstrated that p53 is a multitasking protein [57], shielding the cells against cancer on many levels, including nucleotide excision repair [58–61], base excision repair [62, 63], mismatch repair, DNA double-strand break repair and recombination [64, 65], nonhomologous end joining [66, 67], homologous recombination [68, 69], and interactions with REcQ helicases [70, 71]. The results of the present study align with these studies, i.e., a differential response in cell viability is apparent after treatment with AgNPs that is dependent on the p53 status of the cell lines. It should be mentioned however that the results of the present study are opposite from those reported by Kovacs et al. [37], who have found that p53-expressing U2Os and p53-deficient Saos-2 cells were killed at approximately the same degree, when exposed to AgNPs. The present study demonstrated that the p53-expressing HOS osteosarcoma cells presented a significantly diminished viability when subjected to high concentrations of 7 nm (10 ppm and 5 ppm) and 60 nM (10 ppm) AgNPs, thus suggesting that p53 does play a role in AgNP-mediated cytotoxicity. The putative mechanism is via high oxidative stress leading to the production of increased ROS levels [72]. Excessively high levels of ROS cause damage to essential cell ingredients and structures, such as nucleic acids, proteins, lipids, membranes, and organelles. This is followed by activation of certain processes, leading eventually to apoptosis [73]. The main difference between the Saos-2 and the HOS cells is that the latter expresses the p53 protein. Therefore, it is logical to assume that p53 is the factor that is responsible for the diminished viability of the HOS osteosarcoma cells when exposed to AgNPs at high concentrations. As the levels of ROS in the HOS cells increase due to the encapsulation of AgNPs, there is an interaction between the ROS and p53. A previous study which conducted a microarray examination of cells treated with hydrogen peroxide has found 16 genes, highly responsive to H_2_O_2_, which were targeted by p53 [74]. Increased levels of ROS in HOS cells stimulate certain pathways combining p53 and redox signaling. The levels of ROS play a very important role in the signals that will be initiated, in order for p53 to target certain genes which will determine the fate of the osteosarcoma cell. Two studies have found that p53 suppresses antioxidant genes, and as a result, cellular ROS levels increase, leading to oxidative stress. Specifically, the manganese superoxide dismutase (MnSOD) gene, which encodes an antioxidant enzyme (SOD2) that protects cells from oxidative damage, is suppressed at the promoter level by either p53 activation or by p53 overexpression [75, 76]. However, other antioxidant genes, such as ALDH4 (aldehyde dehydrogenase 4), and PIG12, which is a novel member of the microsomal glutathione S-transferase gene family, have been shown to be concurrently upregulated with p53 overexpression and seem to be like an adaptive response to oxidative stress induced by p53 [77–79]. Increased ROS levels causing oxidative stress lead to mitochondrial lipid degradation, as well as morphological changes, i.e., chromatin condensation and fragmentation, and biochemical alterations, i.e., poly ADP-ribose polymerase (PARP) caspase-mediated degradation, which are definite signs of cellular apoptosis [77].

### 4.3. Effects of Treatment Time on Cell Viability

Time-dependent viability was also observed in the present study. A slight increase in cell viability was noted from 24 to 72 hours for the 7 nm at 10 ppm and 5 ppm and for the 60 nm for the 10 ppm. However, for the big majority of the remaining concentrations (2.5 ppm, 1.25 ppm, 0.625 ppm, and 0.3125 ppm), this trend was not observed. On the contrary, a marked decrease in HOS cell viability was observed from 24 to 72 hours, reaching values to about 60-70% of the control. This finding is in alignment with the results of Kovacs and coworkers, who checked cell viability at 24 and 48 hours [37]. Decreased cell viability may indicate that the release of silver ions from the AgNPs in the lowest concentrations takes more time, but after 24 hours, the silver ion concentrations reach a level which is capable of contributing to the generation of ROS, which finally reach toxic levels and trigger apoptosis. Although Saos-2 cell viability at 72 hours was decreased compared to that of 24 hours, the cell viability values were very close to those of the control.

### 4.4. Effect of AgNPs' Size and Concentration on Cell Proliferation

In addition to cell viability, which was tested with the MTT assay, the BrdU test was used to target the cell proliferation rates of p53-deficient Saos-2 and p53-expressing HOS osteosarcoma cells. As BrdU is incorporated into newly synthesized DNA, it can detect which cells are in the S-phase of the cell cycle [80]. The same trend that was noticed for metabolic activity, measured by the MTT assay, was also noticed when the BrdU assay was employed. A marked difference was noticed between the p53-deficient Saos-2 cells and the p53-expressing HOS cells. In general, cell proliferation of the HOS cells was decreased from the 24-hour to 72-hour time interval. Once again, the 7 nm AgNPs had a marked effect on reduced HOS cell viability at both the 10 ppm and 5 ppm concentrations, while the 60 nm AgNPs presented a noticeable effect at only the highest concentration. A clear effect on reduced Saos-2 cell viability was not noted at either the 7 nm or the 60 nm AgNPs, at any concentration.

Regarding cell proliferation, the results of the present study are not in accordance with those reported by Kovacs et al., who have found that p53-expressing U2Os and p53-deficient Saos-2 cells presented similar proliferation rates (in relation to the control), when exposed to AgNPs [37]. The present study demonstrated that the p53-expressing HOS osteosarcoma cells presented significantly less proliferation compared to p53-deficient Saos-2 cells when subjected to 7 nm AgNPs at 10 ppm and 5 ppm concentrations, and 60 nm at 10 ppm concentration. The diminished cell proliferation noted for all time periods when HOS cells were treated with 7 nm AgNPs at 10 ppm and 5 ppm, and with 60 nm AgNPs at 10 ppm is probably due to DNA damage through the elevation of ROS at toxic levels. Several studies have demonstrated that p53 apoptotic signals lead to activation of caspase [81, 82]. However, the exact mechanism by which this activation occurs is still not well defined. It has also been speculated that mitochondrial cytochrome c (mtCyt c), which is needed for ATP production, is also involved in the apoptotic procedures. Gao and colleagues reported that the cytosolic release of cytochrome c, which activates caspases, and membrane translocation of Bax are both mediated by protein p53 [83]. Caspase-3 in particular is responsible for morphological changes in the nucleus, through cleavage of a variety of substrates, as well as for disintegration of the DNA [82].

In addition to these apoptotic procedures, p53 is responsible for cell cycle arrest, through interaction with protein p21 (WAF1/C1P1), which acts as a signal to halt cell division. p21 binds to cyclin-CDK complexes, which are responsible for promoting the cell cycle. This binding has as a result the inhibition of kinase activity and therefore arrest of the cell cycle. It has been demonstrated that gene p21 has numerous elements mediating p53 binding, activating in this way the gene which is responsible for p21 protein encoding [84, 85].

In general, the results of the present study confirm that lower size AgNPs are more effective at decreasing cell viability than the larger ones. However, in conflict with previously published work [37], we found that AgNP treatment markedly decreased cell viability of p53-expressing HOS cells, but not of p53-deficient Saos-2 cells, suggesting a role for p53 in AgNP-mediated cytotoxicity. Therefore, this study supports the notion that the treatment with AgNPs is more effective if the osteosarcoma cells express protein p53.

## 5. Conclusions

The results of the present study suggest that AgNPs of a smaller diameter (7 nm) are more effective on osteosarcoma cell viability and cell proliferation than those of a bigger diameter (60 nm). Furthermore, a higher concentration of AgNPs is more effective than that of a smaller concentration. The 5 ppm concentration is effective only for the 7 nm AgNPs. Within the 72-hour period, treatment with AgNPs of 7 nm at 5 or 10 ppm is highly effective against p53-expressing osteosarcoma cells, but it is not effective against p53-deficient osteosarcoma cells. Concentrations of less than 5 ppm for the 7 nm silver nanoparticles and less than 10 ppm for the 60 nm silver nanoparticles are not effective. The presence of protein p53 affects significantly the efficacy of AgNPs on osteosarcoma cells.

The results of the present study suggest that the use of AgNPs against certain types of osteosarcoma, which involve the presence of protein p53, seems to be effective. However, preclinical testing is needed to further establish the efficacy and the safety of AgNP use. Other parameters, including the best route of administration, the therapeutic window, pharmacodynamics, pharmacokinetics, and any potential side effects, all need to be established. This *in vitro* study contributes to the growing body of evidence that AgNPs might be a useful addition to the armamentarium of osteosarcoma treatment, but there is much to be done before AgNPs can be shown to be effective in the clinical arena. There is however a glimmer of hope that AgNPs may be translated into a useful treatment in the fight against osteosarcoma.

## Figures and Tables

**Table 1a:** (a) 7 nm

Average diameter (nm)	6.93	Solvent	Deionized water
Standard deviation (%)	19.01	Viscosity (cP)	0.888
Concentration (ppm)	1500	Capping agent type	Organic
Zeta-potential (mV)	—	pH	4.15

**Table 1b:** (b) 60 nm

Average diameter (nm)	59.97	Solvent	Deionized water
Standard deviation (%)	13.44	Viscosity (cP)	0.888
Concentration (ppm)	1710	Capping agent type	Organic
Zeta-potential (mV)	—	pH	4.30

**Table 2a:** (a) MTT

7 nm	Control	10 ppm	5 ppm	2.5 ppm	1.25 ppm	0.625 ppm	0.3125 ppm
24 h	100.00 (±11.73)	4.50 (±0.50)	6.67 (±2.25)	94.39 (±12.36)	110.00 (±10.29)	88.33 (±13.45)	87.00 (±14.77)
48 h	100.00 (±5.42)	4.68 (±0.90)	8.25 (±7.03)	97.02 (±9.70)	95.04 (±23.89)	86.23 (±20.14)	80.20 (±2.10)
72 h	100.00 (±5.22)	10.11 (±2.69)	9.66 (±5.98)	72.93 (±4.93)	65.58 (±1.86)	74.29 (±7.92)	87.70 (±4.00)

**Table 2b:** (b) Average

60 nm	Control	10 ppm	5 ppm	2.5 ppm	1.25 ppm	0.625 ppm	0.3125 ppm
24 h	100.00 (±13.76)	5.00 (±1.20)	85.67 (±5.57)	92.33 (±4.65)	85.11 (±5.11)	80.56 (±1.73)	82.06 (±7.22)
48 h	100.00 (±2.89)	4.84 (±3.05)	77.54 (±6.17)	74.37 (±6.99)	81.39 (±14.76)	77.06 (±18.64)	80.16 (±6.01)
72 h	100.00 (±6.35)	12.88 (±9.85)	57.90 (±2.82)	56.13 (±3.23)	57.28 (±7.64)	71.77 (±11.89)	87.25 (5.65)

**Table 3a:** (a) MTT

7 nm	Control	10 ppm	5 ppm	2.5 ppm	1.25 ppm	0.625 ppm	0.3125 ppm
24 h	100.00 (±10.20)	107.88 (±13.32)	119.61 (±6.57)	121.35 (±7.42)	115.80 (±4.85)	114.31 (±10.05)	106.09 (±9.01)
48 h	100.00 (±12.30)	114.44 (±3.21)	104.02 (±8.26)	105.68 (±7.54)	110.19 (±10.49)	100.61 (±1.07)	91.39 (±4.77)
72 h	100.00 (±3.45)	90.03 (±2.50)	95.71 (±1.36)	101.4 (±3.32)	102.43 (±2.05)	104.02 (±1.34)	101.03 (±1.94)

**Table 3b:** (b) Average

60 nm	Control	10 ppm	5 ppm	2.5 ppm	1.25 ppm	0.625 ppm	0.3125 ppm
24 h	100.00 (±12.56)	102.38 (±7.59)	109.51 (±8.74)	110.45 (±12.50)	109.96 (±11.33)	112.28 (±5.57)	106.19 (±15.02)
48 h	100.00 (±2.46)	91.83 (±16.49)	88.27 (±5.02)	84.27 (±10.55)	100.43 (±5.15)	91.86 (±4.47)	87.29 (±7.33)
72 h	100.00 (±1.23)	81.44 (±3.07)	87.02 (±1.13)	95.80 (±3.93)	98.24 (±4.84)	98.89 (±3.18)	97.57 (±3.62)

**Table 4a:** (a) BRDU

7 nm	Control	10 ppm	5 ppm	2.5 ppm	1.25 ppm	0.625 ppm	0.3125 ppm
24 h	100.00 (±15.70)	1.17 (±0.86)	9.20 (±2.88)	112.47 (±25.79)	96.11 (±18.95)	82.67 (±10.46)	61.35 (±2.94)
48 h	100.00 (±6.68)	0 (±0.06)	2.44 (±1.23)	72.62 (±12.73)	75.68 (±18.66)	81.46 (±29.14)	80.94 (±31.82)
72 h	100.00 (±8.54)	-0.69 (±0.27)	4.06 (±1.66)	65.82 (±28.57)	39.49 (±11.78)	61.99 (±5.45)	71.47 (±14.23)

**Table 4b:** (b) Average

60 nm	Control	10 ppm	5 ppm	2.5 ppm	1.25 ppm	0.625 ppm	0.3125 ppm
24 h	100.00 (±15.70)	5.04 (±5.51)	107.67 (±13.20)	112.89 (±5.62)	91.03 (±4.28)	75.78 (±2.56)	89.00 (±8.63)
48 h	100.00 (±6.68)	1.65 (±2.45)	107.91 (±14.10)	97.75 (±16.07)	68.58 (±13.54)	86.65 (±6.10)	115.76 (±23.11)
72 h	100.00 (±8.54)	4.29 (±4.81)	71.43 (±4.17)	52.65 (±16.85)	59.21 (±9.91)	85.10 (±7.20)	92.33 (±24.92)

**Table 5a:** (a) BRDU

7 nm	Control	10 ppm	5 ppm	2.5 ppm	1.25 ppm	0.625 ppm	0.3125 ppm
24 h	100.00 (±6.45)	108.73 (±3.20)	97.73 (±12.41)	94.23 (±5.28)	89.12 (±0.37)	80.88 (±3.92)	101.99 (±4.68)
48 h	100.00 (±6.62)	59.76 (±16.90)	93.42 (±14.61)	95.31 (±8.78)	91.08 (±0.60)	87.58 (±13.32)	99.91 (±21.24)
72 h	100.00 (±7.07)	83.93 (±9.43)	89.84 (±2.81)	89.75 (±5.69)	90.60 (±1.38)	90.89 (±3.83)	97.02 (±6.91)

**Table 5b:** (b) Average

60 nm	Control	10 ppm	5 ppm	2.5 ppm	1.25 ppm	0.625 ppm	0.3125 ppm
24 h	100.00 (18.91)	102.21 (4.83)	81.25 (21.52)	90.01 (3.52)	83.03 (16.40)	86.82 (21.28)	95.95 (10.43)
48 h	100.00 (6.62)	100.23 (2.60)	99.28 (14.65)	118.66 (2.05)	110.94 (8.61)	108.84 (4.82)	104.26 (6.75)
72 h	100.00 (7.07)	93.12 (11.91)	85.25 (14.54)	91.60 (13.22)	84.21 (0.60)	93.57 (5.98)	104.29 (7.52)

## Data Availability

All data are available upon request.
